# Probiotic Supplementation to Prevent Diaper Dermatitis in Antibiotic-Treated Neonates: Secondary Analysis of a Randomised Controlled Trial

**DOI:** 10.3390/children13081059

**Published:** 2026-08-09

**Authors:** Mojca Železnik, Petra Bratina, Andreja Valcl, Katja Lozar Manfreda, Evgen Benedik, Tanja Obermajer, Bojana Bogovič Matijašić, Aleksander Mahnič, Darja Paro-Panjan, Jana Lozar Krivec

**Affiliations:** 1 Department of Neonatology, University Children’s Hospital, University Medical Centre Ljubljana, 1000 Ljubljana, Slovenia; mojca.zeleznik@kclj.si (M.Ž.); darja.paro@gmail.com (D.P.-P.); 2 University of Ljubljana, Faculty of Medicine, Department of Paediatrics, 1000 Ljubljana, Slovenia; 3 Department of Neonatology, Hospital for Women Diseases and Obstetrics Postojna, 6230 Postojna, Slovenia; 4 Department of Paediatrics, General Hospital Slovenj Gradec, 2380 Slovenj Gradec, Slovenia; 5 University of Ljubljana, Faculty of Social Sciences, 1000 Ljubljana, Slovenia; 6 Department of Gastroenterology, Hepatology and Nutrition, University Children’s Hospital, University Medical Centre Ljubljana, 1000 Ljubljana, Slovenia; evgen.benedik@kclj.si; 7 University of Ljubljana, Biotechnical Faculty, Department of Food Science and Technology, 1000 Ljubljana, Slovenia; 8 University of Ljubljana, Biotechnical Faculty, Institute of Dairy Science and Probiotics, Department of Animal Science, 1230 Domžale, Slovenia; tanja.obermajer@bf.uni-lj.si (T.O.); bojana.bogovicmatijasic@bf.uni-lj.si (B.B.M.); 9 Department for Microbiological Research, National Laboratory for Health, Environment and Food, 2000 Maribor, Slovenia; alemah1@nlzoh.si; 10 University of Maribor, Faculty of Medicine, 2000 Maribor, Slovenia

**Keywords:** antibiotics, breastfeeding, diaper dermatitis, gut microbiota, *Limosilactobacillus reuteri*

## Abstract

**Highlights:**

**What are the main findings?**

- Supplementation with *Limosilactobacillus reuteri* DSM 17938 during and after antibiotic treatment did not reduce the incidence of diaper dermatitis in neonates.
- Probiotic supplementation did not significantly alter gut microbiota composition or diversity.

**What are the implications of the main findings?**

- Routine use of *Limosilactobacillus reuteri* DSM 17938 for the prevention of diaper dermatitis in antibiotic-treated neonates is not supported by these findings.
- Further adequately powered studies are needed to evaluate strategies for the prevention of diaper dermatitis in antibiotic-treated neonates.

**Abstract:**

**Background/Objectives:** Diaper dermatitis (DD) is a common dermatological condition that is often exacerbated by antibiotics. Probiotics may restore microbiota balance and influence the incidence of DD. This study aimed to evaluate whether 6 weeks of *Limosilactobacillus* (*L.*) *reuteri* DSM 17938 supplementation during and after antibiotic treatment affects the incidence of DD. **Methods:** A secondary analysis of a randomised, double-blind, placebo-controlled trial that included 89 term neonates treated with antibiotics during the first 3 weeks of life was conducted. Neonates were assigned to receive either a probiotic supplement or a placebo for a total of 6 weeks. DD incidence, onset of DD, and skin care data were collected for 87 infants. Faecal samples were collected 6 weeks after study enrolment to analyse the composition of gut microbiota. **Results:** Twenty-four (28%) infants developed DD, of whom six (25%) had fungal DD. There was no significant difference in the incidence of DD or fungal DD between the probiotic and placebo groups (30% vs. 25% and 7% in both groups, respectively). Probiotic supplementation had no significant effect on the gut microbiota composition or diversity. In an exploratory binary logistic regression analysis based on six fungal DD events, probiotic supplementation was not associated with fungal DD, whereas breastfeeding was associated with lower odds of fungal DD; however, the overall model was not statistically significant, and this finding should be interpreted with caution because of the small number of events. **Conclusions:** In this exploratory secondary analysis, supplementation with *L. reuteri* DSM 17938 was not associated with a reduced incidence of DD or alterations in gut microbiota composition in antibiotic-treated neonates. Breastfeeding was associated with lower odds of fungal DD; however, this finding should be confirmed in larger, adequately powered studies.

## 1. Introduction

Diaper dermatitis (DD) is a common dermatological problem in infancy [1]. The term is used to describe any of the various inflammatory reactions of the skin in the diaper area [2]. The development of DD is multifactorial; infrequent diaper changes lead to persistent wetness, maceration, and damage to the stratum corneum [3]. In addition to humidity and friction, DD is associated with a combination of other factors, such as the presence of irritants in urine and faeces, increased pH [3], and disrupted skin microbiota [4].

The neonatal gut microbiome undergoes rapid development during the first weeks of life and is strongly influenced by early-life exposures, particularly antibiotic use and infant feeding, which shape microbial colonisation and immune development [5,6].

Antibiotics are often associated with increased severity and frequency of DD [7,8,9]. Antibiotics have been associated with increased numbers of *Candida albicans* in the genital area [7]. Additionally, perinatal antibiotics disrupt initial colonisation, leading to dysbiosis [10], which can progress to antibiotic-associated diarrhoea [2,8]. The incidence of DD is higher in patients with diarrhoea, as they have increased lipase and protease activity in the faeces due to the acceleration of gastrointestinal passage and a shorter transit time, which in turn compromises the integrity of the stratum corneum [2]. Broad-spectrum antibiotics and diarrhoea are frequently reported in the medical history of patients with DD [2,8].

Some studies have shown that exclusively breastfed infants have a lower risk of DD because their faeces are less irritating and has a lower urease content and lower protease and lipase activity, resulting in a significantly lower faecal pH than that of formula-fed infants [2]. This difference is closely linked to the abundance of *Bifidobacterium* in breastfeeding infants [11]. However, previous research has shown that early antibiotic exposure could affect the composition of the gut microbiota, resulting in significantly lower levels of *Bifidobacterium* [12], which may impact the faecal pH and microbiota balance.

Probiotics have been proposed as a strategy to restore gut microecological balance following antibiotic exposure and improve intestinal colonisation [13,14]. Recent evidence indicates that probiotics may mitigate antibiotic-associated adverse effects by maintaining gut microbial homeostasis, strengthening intestinal barrier function, and modulating host immune responses [15]. However, probiotic effects are strain-specific and cannot be generalised across different probiotic species or strains. Their efficacy also depends on dosage, duration of supplementation, timing of administration, and characteristics of the target population [16]. Lactobacilli promote intestinal homeostasis through multiple mechanisms, including competitive exclusion of pathogenic microorganisms, production of antimicrobial compounds, enhancement of intestinal epithelial barrier integrity, reduction in intestinal pH, and modulation of immune responses [17]. This study aimed to investigate whether 6 weeks of supplementation with the probiotic *Limosilactobacillus* (*L.*) *reuteri* DSM 17938 during and after antibiotic treatment in neonates influenced the occurrence of DD. We hypothesised that the administration of probiotics to infants treated with antibiotics in the early neonatal period would beneficially influence gut microbiota composition and diversity, resulting in a lower incidence of DD compared with the placebo group.

## 2. Materials and Methods

### 2.1. Study Design and Aim

This study was a randomised, double-blind, placebo-controlled trial, (ClinicalTrials.gov; NCT02865564) conducted at three Slovenian healthcare centres: Department of Neonatology, University Children’s Hospital Ljubljana, University Medical Centre Ljubljana; Department of Neonatology, Hospital for Women’s Diseases and Obstetrics Postojna; and Department of Paediatrics, General Hospital Slovenj Gradec. A detailed methodology is described in Lozar Krivec et al. (2024) [18]. This article reports on a predefined secondary objective of the trial: evaluating the influence of *L. reuteri* DSM 17938 supplementation during and after antibiotic treatment on the incidence of DD, which was assessed as part of an online parental questionnaire.

### 2.2. Participants

Neonates treated with antibiotics for suspected early or late neonatal sepsis were eligible. The inclusion criteria were antibiotic treatment for ≥5 days, age <21 days at the initiation of antibiotic treatment, and term birth (gestational age of ≥37 weeks). Participants received ampicillin or flucloxacillin plus gentamicin for 7 to 10 days, according to hospital antibiotic protocol. Written informed consent from parents was obtained prior to inclusion. The exclusion criteria included prematurity, birth weight <2500 g, congenital malformations/syndromes, perinatal hypoxia (Apgar score <5 at 5 min), and the use of probiotics before/during the study. The aim was to enrol 100 neonates, randomised 1:1 to the study or placebo group.

### 2.3. Study Procedure—Randomisation and Intervention

A computer-generated randomisation scheme ensured group balance. Allocation to the probiotic or placebo group was concealed from all investigators and participants and was revealed after the last follow-up at one year of infant age. The probiotic group received five drops daily of the active study product (freeze-dried *L. reuteri* DSM 17938, 1 × 10^8^ CFU/day); the placebo group received 5 drops daily of the placebo study product (containing an identical formulation in all respects except for the probiotic bacteria). Supplementation started on the first day of antibiotic treatment and continued for 6 weeks, during hospitalisation and in the home environment. The study product was administered by nursing staff in the hospital and by parents at home. Prior to discharge, parents received comprehensive instructions on the correct administration of the study product. The products were provided free of charge by BioGaia (Stockholm, Sweden). BioGaia was not involved in the planning, design or conduct of the study, or in the analysis or interpretation of the data.

### 2.4. Data Collection and Follow-Up

Demographic and clinical data were collected from medical documentation. DD was defined as an erythematous rash with or without scaling, swelling, or scattered erythematous papules and ulcers. Parents received detailed written instructions regarding the recognition and reporting of DD, and in cases of diagnostic uncertainty, the child was examined and the diagnosis confirmed by the medical professional. The definition of fungal DD encompassed cases that had been clinically diagnosed as fungal (deep red rash, maceration in inguinal folds, satellite papules or pustules surrounding the main rash) and treated with an antifungal skin cream prescribed by a physician, with subsequent clinical improvement [19]. No microbiological confirmation was performed. Data on DD and skincare cream use were collected at the end of antibiotic treatment during hospitalisation and 4 weeks later by an online follow-up questionnaire completed by the parents. All parents were instructed to change the diaper every 3 h and to use barrier cream at each change. A text message and email, both containing a link to the online questionnaire and an identifier code, were dispatched one day prior to remind parents to complete it. Other clinical data (gastrointestinal signs, daily crying and sleeping time, mode of feeding, possible allergies and dietary supplementation, antibiotic use and infectious diseases during the observational period, defaecation frequency, week of DD occurrence after antibiotic treatment, cream used for DD, compliance with the study product) were collected via an online questionnaire.

### 2.5. Faecal Sample Collection, 16S Amplicon Sequencing, and Real-Time PCR Analysis

Faecal samples were collected in sterile containers 6 weeks after enrolment. The detailed protocols for sample collection, storage, microbial DNA extraction, and analysis are described in Lozar Krivec et al. (2024) [18]. Briefly, 16S rRNA gene amplicon sequencing targeting the V3–V4 variable region was performed using the Illumina MiSeq platform (San Diego, CA, USA) (2 × 300 bp). Sequence data were quality-filtered and clustered into zero-radius operational taxonomic units (zOTUs) using USEARCH v11.0.667 (Stanford, CA, USA), and taxonomy was assigned using the RDP trainset v18. The sequencing data are available from the NCBI database under accession number PRJNA954698.

Additionally, real-time PCR targeting five bacterial groups (total bacteria, *Bifidobacterium*, *Enterobacteriaceae*, *Lactobacillaceae*, and *L. reuteri* DSM 17938) was performed using KAPA SYBR Fast Master Mix Universal (KapaBiosystems, Woburn, MA, USA) on a Bio-Rad CFX96 Touch real-time PCR instrument (Bio-Rad, Hercules, CA, USA). Sample concentrations (expressed as target copies per gram of faeces) and corresponding limits of detection (LODs), along with reaction parameters, were calculated as described by Obermajer et al. [20].

### 2.6. Statistical Analysis

The probiotic and placebo groups were compared in terms of DD incidence, skincare cream use, compliance with the study product, target intestinal bacteria, and alpha and beta microbial diversity. Clinical data were analysed using IBM SPSS Statistics, version 29 (IBM Corporation, Armonk, NY, USA). Relative sample frequencies and absolute risk differences are reported for DD incidence and skincare cream use in the probiotic and placebo groups, together with the 95% confidence interval for the difference in percentages for the DD incidence (using Wald or Newcomb–Wilson method). Binary logistic regression was implemented to assess the association between probiotic supplementation and the occurrence of DD and fungal DD only while adjusting for potential confounding variables, including gender, mode of delivery, and breastfeeding. Variables included in the models were selected based on previously reported risk factors for DD and fungal DD and their clinical relevance. Given the limited number of fungal DD events, the regression model for fungal DD was considered exploratory. The low event-to-variable ratio increased the risk of overfitting and unstable estimates; therefore, results from this model should be interpreted with caution. Model assumptions were assessed by evaluating multicollinearity among predictor variables, and overall model fit was evaluated using the Hosmer and Lemeshow goodness-of-fit test.

Analysis and visualisation of the microbiome data were performed in R (v4.2.2, R Core Team, 2022, Vienna, Austria) by the ‘vegan’ 2.6.-4, ‘picante’ 1.8.2 and ‘ggplot2’ 4.0.0. packages. For multiple comparisons, the Benjamini–Hochberg procedure was applied to control the false discovery rate. A *p*-value < 0.05 was considered indicative of statistical significance.

## 3. Results

Initially, 95 neonates were enrolled, and 89 completed the intervention period (3 parents withdrew consent during the antibiotic treatment, 1 stopped the intervention after 2 weeks, and 2 introduced additional probiotics). Among the 89 participants (probiotic: n = 44, placebo: n = 45), 87 completed the online questionnaire after 4 weeks and were included in this analysis. Faecal samples were collected from 85 participants 6 weeks after enrolment. The probiotic and placebo groups were comparable in terms of clinical and demographic characteristics, except for the birth measurements, but the difference was not clinically important. Compliance with the study product during the four-weeks post-discharge follow-up period was high, with 88.4% of participants in the probiotic group and 79.1% in the placebo group taking the product daily (*p* = 0.243), and the remaining participants taking it almost every day. At enrolment, 59.1% of neonates in the probiotic group and 51.1% in the placebo group were exclusively breastfed (*p* = 0.751). After 4 weeks, this decreased to 54.5% and 45.5% (*p* = 0.423), respectively, while exclusive formula feeding increased from 11.4% to 15.9% in the probiotic group and from 13.3% to 27.2% in the placebo group. Detailed information on the clinical characteristics is provided in Lozar Krivec et al. (2024) [18].

Twenty-four out of 87 infants developed DD (28%, 95% CI 0.18–0.36). Two neonates had DD during hospitalisation and in the first week after hospitalisation, so we did not count them twice. Fourteen (63.6%) infants developed DD in the first week, 5 (22.7%) in the second week, and 3 (13.6%) in the third week after antibiotic treatment. Confidence intervals for the difference showed no significant differences in DD incidence observed between groups (Table 1).

A healing cream containing 15.25% zinc oxide and lanolin was the most commonly used (Table 2). In Slovenia, antifungal creams are available upon doctor prescription. Six infants with DD used antifungal cream (1 infant used 2 types of antifungal cream), suggesting the occurrence of fungal DD. This corresponded to a 25% incidence of fungal DD among infants with DD and a 7% incidence among the entire study population. Confidence intervals for the difference showed no significant difference in the incidence of fungal DD between the groups (Table 1).

### 3.1. Diaper Dermatitis in the Context of Other Risk Factors

Analysis of the effect of probiotic supplementation on DD or fungal DD while controlling for other risk factors (gender, mode of delivery, breastfeeding) with binary logistic regression models revealed no statistically significant effect of the probiotic supplementation (*p* = 0.543), nor of any other risk factor (*p* = 0.954 for the goodness-of-fit test). However, in an exploratory model based on six fungal DD events only, breastfeeding was associated with lower odds of fungal DD (OR = 0.056; 95% CI 0.003; 1.122); however, the overall model was not statistically significant (*p* = 0.965), and the estimate was highly imprecise. There was no statistical difference in the proportion of infants with >4 bowel movements per day between infants with (22/24, 91.7%) and without (55/63, 87.3%; *p* = 0.72) DD. The absolute difference in proportions was 4.4% (95% CI −16.4% to 14.2%).

### 3.2. Diaper Dermatitis-Associated Alterations in the Gut Microbiota Composition

**Figure 1:**
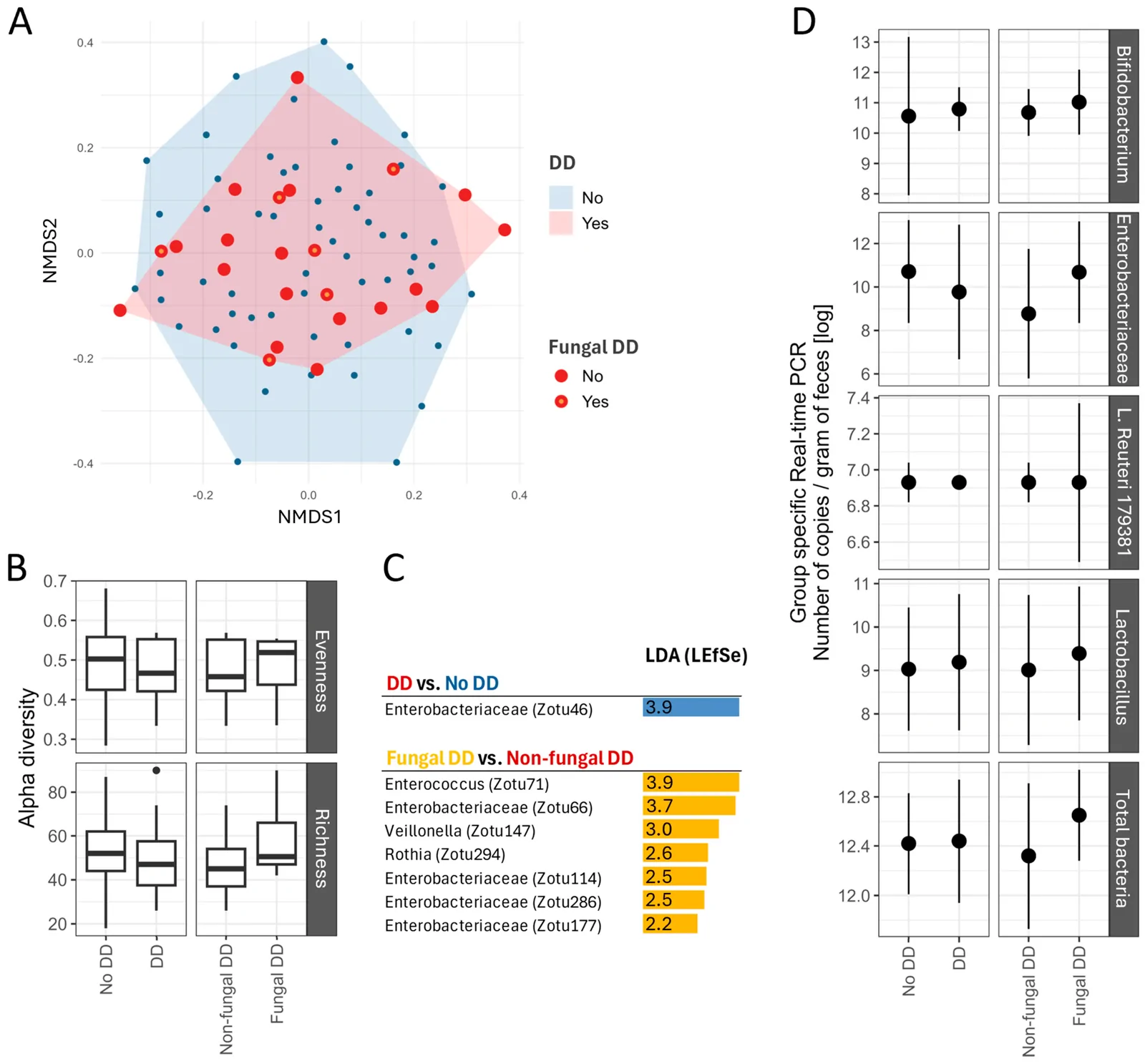
Diaper dermatitis-associated alterations in the gut bacterial community structure. (**A**) NMDS plot based on Bray–Curtis distances, colour-coded by probiotic group affiliation. (**B**) Distributions of alpha diversity metrics (community evenness and richness), comparing individuals with and without DD and further stratified by fungal DD versus non-fungal DD. (**C**) Differentially abundant taxa identified using the LEfSe algorithm. The results are presented for two comparisons: DD vs. no DD and fungal DD vs. non-fungal DD. (**D**) Group-specific real-time PCR measurements presented as target copy numbers per gram of faeces (median ± IQR). The results are shown for two comparisons: DD vs. no DD and fungal DD vs. non-fungal DD. Abbreviation: DD, diaper dermatitis.

Bacterial community analysis revealed no significant differences between the probiotic and placebo groups. Detailed data are presented in the study by Lozar Krivec et al. [18]. Furthermore, the beta diversity measures did not significantly differ between individuals with and without DD (AMOVA *p* = 0.636; ANOSIM *p* = 0.836), or between fungal DD versus non-fungal DD cases (AMOVA *p* = 0.974; ANOSIM *p* = 0.988) (Figure 1A). Similarly, no significant differences were detected in the alpha diversity metrics, including richness (number of observed taxa; *p* > 0.12 for all comparisons) and community evenness (*p* > 0.28 for all comparisons) (Figure 1B).

At the population level, several differentially abundant taxa were identified; however, none remained statistically significant after false discovery rate correction (Benjamini–Hochberg procedure, FDR > 0.05). Notably, an exploratory trend toward increased abundance of several taxa within the family *Enterobacteriaceae* was observed in infants with fungal dermatitis (Figure 1C). This trend was not supported by *Enterobacteriaceae*-specific real-time PCR, suggesting that the observed signal may be driven by select taxa rather than the family as a whole (Figure 1D). Finally, no significant differences between the study groups were detected in terms of the total bacterial load, *L. reuteri* DSM 17938 (probiotic strain) abundance, or total levels of biologically relevant *Lactobacilli* and *Bifidobacteria*, as determined by absolute quantification with group-specific real-time PCR (Figure 1D).

## 4. Discussion

To our knowledge, this is the first randomised controlled trial evaluating the effect of *L. reuteri* DSM 17938 supplementation on the development of DD in infants receiving antibiotics. Our results indicate that probiotic supplementation with *L. reuteri* DSM 17938 had no effect on the incidence of DD. Furthermore, few DD-associated alterations in the gut microbiota were observed. These findings contrast with those of several studies suggesting that probiotics might influence both the skin and gut microbiota and potentially reduce the incidence of DD [21,22,23].

In our study population, the incidence of DD was 28%. The reported incidence of DD varies widely across studies. Two large studies reported incidences of 25% in the first 4 weeks of life [24] and 48% in infants aged 1–6 months [25]. Antibiotics are known risk factors for DD, presumably acting by influencing the skin and gut microbiota and increasing the incidence of diarrhoea [26,27], although data on DD in antibiotic-treated infants are scarce. A study published in 1988 reported a 15% incidence of DD associated with amoxicillin treatment [7].

We hypothesised that 6 weeks of probiotic supplementation would mitigate antibiotic-induced alterations in the microbiota and reduce the incidence of DD. However, we did not find a significant difference in incidence of DD between the probiotic and control groups. Similarly, the comparison of the gut microbiota 6 weeks after study enrolment revealed no significant difference between the groups [18].

All neonates in our study received systemic antibiotic treatment, which is known to disrupt the developing neonatal gut microbiota and alter its composition during a critical period of microbial colonisation [28]. Such alterations may contribute to dysbiosis and antibiotic-associated diarrhoea, both of which have been implicated in the development of DD [2]. Neonates are commonly exposed to combination antibiotic regimens, such as a β-lactam antibiotic in combination with gentamicin. Consequently, most clinical studies have evaluated the effects of the combined regimen rather than the individual effects of each antibiotic. Treatment with this antibiotic combination has been associated with a reduced abundance and diversity of beneficial bacteria, particularly *Bifidobacterium* spp., delayed colonisation by *Bacteroidetes*, and the relative expansion of opportunistic taxa, including *Enterococcus* and *Klebsiella* [29]. Although probiotics have been proposed as a strategy to mitigate antibiotic-associated adverse effects, their efficacy depends on the administered strain and clinical context, and current evidence suggests that responses may vary according to the extent of microbiota disruption and timing of administration [28,30]. Therefore, the lack of a significant effect of *L. reuteri* DSM 17938 in our study may be related to the extent of antibiotic-induced microbiota disruption, the timing of probiotic administration during antibiotic treatment, or the low intestinal detection rate of *L. reuteri* DSM 17938 observed in our cohort. High antibiotic pressure was reported not to affect the general *L. reuteri* DSM 17938 intestinal colonisation unless the specific antibiotics were applied [31]. *L. reuteri* DSM 17938 was reported to be susceptible to gentamicin (but not ampicillin) [32].

A recent study by Teufel et al. analysed the skin microbiota in the diaper area and revealed no change in bacterial diversity but highlighted a shift in abundance with increasing DD severity. Compared with unaffected skin, more severely affected skin had a greater percentage of faecal coliform bacteria, such as *Enterococcus,* and a lower percentage of the overall predominant *Staphylococcus* species [21]. Another study in infants aged 0–2 years revealed that the contents of *Ligilactobacillus ruminis* (formerly *Lactobacillus ruminis*), *Bifidobacterium longum,* and *Clostridium butyricum,* as well as the skin probiotic bacteria *Staphylococcus epidermidis*, significantly decreased in DD lesions. Moreover, the *Lactobacillaceae* and *Bifidobacterium* populations began to recover after DD was resolved. These findings suggest that the gut microbiota may change dynamically during the course of DD, influencing skin dysbiosis in the diaper area and supporting the potential role of probiotics in preventing and resolving DD [22]. However, our findings did not support this association, as no significant differences in gut microbiota composition, bacterial community structure, richness, or diversity were observed between infants with and without DD. A large consumer survey revealed a 72% reduction in the occurrence of DD in infants with *Bifidobacterium infantis* EVC001 supplementation, although methodological constraints, such as the lack of a placebo group, prevented definitive conclusions [23]. Importantly, probiotic effects are strain-specific and cannot be generalised across different probiotic species or strains. Therefore, the absence of a significant benefit observed with *L. reuteri* DSM 17938 in our study should not be interpreted as evidence that other probiotic strains would be similarly ineffective in preventing DD. To date, most research has focused on probiotics’ potential benefits on atopic dermatitis [33], while their role in DD remains less explored.

Antibiotic treatment leads to an increased incidence of *C. albicans* in the gut and diaper area [7]. Brook reported a more than 14-fold increase in *C. albicans* density in the diaper area following amoxicillin therapy [34], and a positive correlation between colonisation density and DD severity was found [9]. However, some healthy infants are colonised with *C. albicans* without any symptoms [9]. Notably, neonates with fungal dermatitis showed modest, largely observational shifts in gut microbiota composition, including a trend toward increased abundance of several different *Enterobacteriaceae* taxa. However, these differences were statistically significant only before false discovery rate correction and did not remain significant after correction. This pattern may nevertheless suggest dysbiosis associated with fungal overgrowth, a phenomenon well-documented in adults but still poorly understood in paediatric populations [35,36].

In our study, the highest incidence of DD was observed during the first week after the completion of antibiotic treatment, supporting the possible short-term effects of antibiotics on the microbiota or the frequency of defaecation. However, our study revealed no difference in the patterns of defaecation between the probiotic and control group or the group with or without DD. In contrast, a study by Paramita and Paramita revealed that diarrhoea duration was correlated with DD severity [37].

Studies on breastfeeding and DD have reported conflicting results. Two large studies reported a greater incidence of DD in breastfed infants during the first weeks of life, possibly due to more frequent defaecation, which is influenced by the composition of breast milk, including the presence of oligosaccharides and its whey-dominant protein ratio [24,38]. However, other studies suggest a protective effect, as exclusively breastfed infants have less irritating faeces than formula-fed infants [2,39]. Furthermore, research indicates that breastfeeding not only helps to prevent DD, but also supports the healing process and reduces inflammation due to the presence of many active biomolecules in human milk [40]. In our study, an exploratory logistic regression analysis suggested an association between breastfeeding and lower odds of fungal DD. However, this finding was based on only six fungal DD events, the overall model was not statistically significant, and the estimate should therefore be interpreted with caution. This observation should be considered hypothesis-generating and requires confirmation in adequately powered studies. In addition, fungal DD was diagnosed clinically and should be confirmed microbiologically in future studies.

Our study has several potential limitations. First, given that DD was a predefined secondary outcome of the trial and the sample size was not powered for this analysis, our findings should be interpreted as exploratory and hypothesis-generating rather than confirmatory. Therefore, the possibility of a type II error cannot be excluded, and the absence of statistically significant differences should not be interpreted as evidence of the absence of potential effect. Furthermore, the exploratory logistic regression analysis of fungal DD was based on only six events, resulting in a low event-to-variable ratio and an increased risk of unstable estimates and model overfitting. Additionally, we did not collect faecal samples during the active phase of DD, and the analysis was conducted on faecal samples collected after the completion of supplementation with the probiotic or placebo. This fact makes it unclear whether transient microbiota changes were the cause of DD and whether this reduced the ability to detect potential differences in the gut microbiota associated with DD and probiotic supplementation. The infant microbiome is characterised by significant variability, and transient changes in the microbiota may have occurred earlier. Therefore, this temporal mismatch represents an important limitation of our study. Furthermore, the acquisition of additional data pertaining to the skin microbiota in regions affected by DD could contribute to a better understanding of the microbiota factors involved in the development of DD. In addition, the low colonisation rate of *L. reuteri* DSM 17938 in the probiotic group may have affected the trial results. Although the detection of DNA from *L. reuteri* DSM 17938 in faeces could not be equated with successful intestinal colonisation, it is also possible that infants did not receive a dose of *L. reuteri* DSM 17938 high enough to have a detectable effect. The incidence of DD, as well as compliance with the study product (except during hospitalisation) were determined through parental self-reports and may be subject to bias due to the subjective interpretation of skin health, although standardised definition and medical professional confirmation in uncertain cases were applied to minimise this bias. Additionally, as mentioned, the diagnosis of fungal DD in our study was based on clinical examination without microbiological confirmation, which may have led to misclassification. Furthermore, DD severity was not assessed using a validated clinical scoring system, which limited the objective assessment of disease severity.

Given our results, it would be valuable to investigate the prevalence of microbiologically confirmed fungal DD, the interplay between skin and gut microbiota, and longitudinal changes in both clinical presentation and microbiota composition throughout the course of DD in antibiotic-treated and healthy infants.

## 5. Conclusions

This study aimed to evaluate whether supplementation with *L. reuteri* DSM 17938 during and after antibiotic treatment could influence the incidence of DD and modulate gut microbiota composition. In this secondary analysis, we found no significant effect of *L. reuteri* DSM 17938 on DD incidence or gut microbiota composition in antibiotic-treated infants. An exploratory association between breastfeeding and lower odds of fungal DD was observed; however, this finding requires confirmation in adequately powered studies. Our findings apply to term neonates receiving the studied antibiotic regimen and should not be generalised to preterm infants or other neonatal populations with different patterns of antibiotic exposure. Further research is warranted to elucidate the role of the gut microbiota in the pathogenesis and prevention of DD.

## Figures and Tables

**Table 1:** Incidence of diaper dermatitis.

Variables	Probiotic	Placebo	Total	Absolute Risk Difference (% Points)	95% CI for Difference in %
DD during hospitalisation, n (%) ^1^, n = 88	1 (2.3)	2 (4.5)	3 (3.4)	−2.2	−9.8; 5.3
DD 4 weeks following antibiotic treatment, n (%) ^2^, n = 87	13 (30.2)	10 (22.7)	23 (26.4)	7.5	−11.0; 26.0
DD cumulative, n (%) ^2^, n = 87	13 (30.2)	11 (25.0)	24 (27.6)	5.2	−13.5; 24.0
Fungal DD, n (%) ^2^, n = 87	3 (7.0)	3 (6.8)	6 (6.9)	0.2	−10.5; 10.8

Abbreviations: DD, diaper dermatitis; CI—confidence interval. ^1^—Data for 88 participants, 44 in the placebo group and 44 in the probiotic group. ^2^—Data for 87 participants, 44 in the placebo group and 43 in the probiotic group.

**Table 2:** Skincare cream used for diaper dermatitis prevention by providers in the home environment.

Cream Used	Probiotic (n = 13)	Placebo (n = 10)	Total (n = 23) *	Absolute Risk Difference (% Points)	95% CI for Difference in %
Clotrimazole, n (%)	3 (23)	3 (30)	6 (26)	−7.0	−0.407; 0.264
Miconazole, n (%) ^1^	1 (8)	0 (0)	1 (4)	8.0	−0.208; 0.333
Healing cream (15.25% zinc oxide and lanolin), n (%) ^1^	5 (38)	5 (50)	10 (43)	−12.0	−0.451; 0.255
Barrier baby cream, n (%) ^1^	3 (23)	3 (30)	6 (26)	−7.0	−0.407; 0.264
Dexpanthenol cream, n (%) ^1^	3 (23)	2 (20)	5 (22)	3.0	−0.313; 0.338
Other creams, n (%) ^1^	5 (38)	5 (50)	10 (43)	−12.0	−0.451; 0.255

Abbreviations: CI—confidence interval. ^1^—Fifteen participants used more than one cream. *—For one participant, we did not have data about the cream used.

## Data Availability

The 16S rRNA gene sequencing data are publicly available in the NCBI database under accession number PRJNA954698. Other datasets generated and analysed during the current study are available from the corresponding author on reasonable request.

## Abbreviations

The following abbreviations are used in this manuscript:

DD	Diaper Dermatitis
(*L.*)	*Limosilactobacillus*
